# Aberrant Topological Patterns of Structural Covariance Networks in Cognitively Normal Elderly Adults With Mild Behavioral Impairment

**DOI:** 10.3389/fnana.2021.738100

**Published:** 2021-09-29

**Authors:** Jun Shu, Qiang Qiang, Yuning Yan, Yiqing Ren, Wenshi Wei, Li Zhang

**Affiliations:** Department of Neurology, Cognitive Disorders Center, Huadong Hospital, Fudan University, Shanghai, China

**Keywords:** mild behavioral impairment, structural covariance network, gray matter volume, small-world, neuroimaging

## Abstract

Mild behavioral impairment (MBI), characterized by the late-life onset of sustained and meaningful neuropsychiatric symptoms, is increasingly recognized as a prodromal stage of dementia. However, the underlying neural mechanisms of MBI remain unclear. Here, we examined alterations in the topological organization of the structural covariance networks of patients with MBI (*N* = 32) compared with normal controls (*N* = 38). We found that the gray matter structural covariance networks of both the patients with MBI and controls exhibited a small-world topology evidenced by sigma value larger than one. The patients with MBI had significantly decreased clustering coefficients at several network densities and local efficiency at densities ranging from 0.05 to 0.26, indicating decreased local segregation. No significant differences in the characteristic path length, gamma value, sigma value, or global efficiency were detected. Locally, the patients with MBI showed significantly decreased nodal betweenness centrality in the left middle frontal gyrus, right inferior frontal gyrus (opercular part), and left Heschl gyrus and increased betweenness centrality in the left gyrus rectus, right insula, bilateral precuneus, and left thalamus. Moreover, the difference in the bilateral precuneus survived after correcting for multiple comparisons. In addition, a different number and distribution of hubs was identified in patients with MBI, showing more paralimbic hubs than observed in the normal controls. In conclusion, we revealed abnormal topological patterns of the structural covariance networks in patients with MBI and offer new insights into the network dysfunctional mechanisms of MBI.

## Introduction

Mild behavioral impairment (MBI) is a neurobehavioral syndrome characterized by the late-life onset of sustained and meaningful neuropsychiatric symptoms (NPSs) of any severity that other formal medical and psychiatric nosologies cannot account for Mortby et al. (2018). MBI can be divided into five subcategories: decreased motivation and drive, affective/emotional dysregulation, impulse dyscontrol, social inappropriateness, and abnormal perception or thought content (Ismail et al., 2016). Growing evidence suggests that the presence of MBI in older individuals is associated with a greater risk of cognitive decline and dementia than in those without MBI. A large-scale study on cognitively normal individuals showed that individuals with MBI had significantly worse cognitive performance at baseline and a significantly greater decline in attention and working memory over 1 year (Creese et al., 2019). Another longitudinal study on cognitively normal individuals reported that of those who progressed from being normal to MCI or dementia, more than 59% developed NPSs before the diagnosis of any cognitive disorder (Wise et al., 2019). A recent study involving community-dwelling adults showed an association of apathy with an increased risk of developing probable dementia (Bock et al., 2020). Therefore, MBI is becoming increasingly recognized as a prodromal stage of dementia. However, the underlying neural mechanisms of MBI remain unclear.

Structural covariance analysis is a powerful method for investigating large-scale structural brain networks, also referred to as structural covariance networks. This method is widely applied based on a critical assumption that the morphological traits of interconnected brain regions will covary because of coordinated neurodevelopmental influences (Alexander-Bloch et al., 2013). A growing body of literature has demonstrated disrupted covariance between regions as a result of various brain diseases, such as Alzheimer’s disease (AD) (Supekar et al., 2008; Yao et al., 2010; Dai and He, 2014), schizophrenia (Spreng et al., 2019), depression (Neufeld et al., 2020), amyotrophic lateral sclerosis (Zhang et al., 2019), and epilepsy (Li et al., 2020). However, to our knowledge, no study has investigated the structural covariance networks in patients with MBI.

In general, three experimental approaches, which include graph analysis, seed analysis and principal component analysis can be used to reveal structural co-variance networks. Among these, graph theory has been widely employed to characterize the global and local topological characteristics of human brain networks (Lo et al., 2011). Growing evidence suggests that changes in the topological organization of brain networks are associated with impairment of cognitive or behavioral functions (Stam, 2014; Mears and Pollard, 2016; Liao et al., 2017). A retrospective study constructed the resting-state functional networks from AD Neuroimaging Initiative (ADNI) database and demonstrated significantly decreased characteristic path length and increased global efficiency in MCI using graph theory methods (Zhang et al., 2020). Another study reported that individuals with subjective cognitive decline (SCD) exhibited higher nodal degree centrality and lower nodal betweenness in the bilateral hippocampus compared to the healthy controls, which indicated a compensatory mechanism of the functional connectome underlying SCD (Chen et al., 2020). A recent study using graph theory methods demonstrated that distinct changes in functional connectivity at the global and local network levels were associated with different NPS clusters in patients with AD (Chang et al., 2020). Moreover, another recent study reported that alterations in structural network measures in AD-spectrum patients were associated with impaired memory function and pathological biomarkers of AD (Sheng et al., 2021). Structural covariance network analysis using graph theory methods may offer novel insights into the neural mechanisms underlying MBI at the network level.

In the present study, we applied graph theory to investigate the topological abnormalities of structural covariance networks based on gray matter (GM) morphology in cognitively normal older adults with MBI (the MBI group) compared with those without MBI (the normal control group). In addition, some global network parameters, such as small-world parameters, local and global efficiency, and a regional network parameter, i.e., nodal betweenness centrality, were evaluated to describe the topological organization of the structural covariance networks of the two groups. We hypothesized that compared to the normal control group, the MBI group would exhibit aberrant topological patterns of structural covariance networks evidenced by altered global network parameters, altered regional network parameters, or divergent hub distributions.

## Materials and Methods

### Subjects

Seventy cognitively normal older participants (32 patients with MBI, 38 normal controls) aged ≥50 years were recruited from the Department of Neurology at Huadong Hospital, Shanghai, China (from August 2018 to June 2020). This study was approved by the ethics committee of Huadong Hospital (2019K145), and informed consent was obtained from all participants. All participants received clinical, neurological, and neuropsychological assessments and brain magnetic resonance imaging (MRI) examinations. The neuropsychological assessment battery included the Clinical Dementia Rating scale (CDR), Mini-Mental State Examination Scale (MMSE), Geriatric Depression Scale (GDS), Self-rating Anxiety Scale (SAS), and Chinese version of the MBI Checklist (MBI-C). The MBI-C is a validated and efficient MBI case ascertainment tool (Ismail et al., 2017). Moreover, two previous studies determined cutoff points for the MBI-C in detecting MBI in individuals with MCI (Mallo et al., 2018a) and subjective cognitive decline (SCD) (Mallo et al., 2018b). Cui et al. (2019) demonstrated that the Chinese version of the MBI-C has high reliability and validity and could replace the NPI-Q for AD dementia screening in the Chinese population. All participants had a CDR score of 0 and met the following MMSE scores based on education level: (1) middle school level and above, >24 points; (2) primary school level (education years > 6), >20 points; and (3) illiterate, >17 points, and their Clinical Dementia Rating Scale total combined score = 0. The diagnosis of MBI was made based on the ISTAART-AA MBI criteria (Ismail et al., 2016) and an MBI-C score > 8. The normal controls did not meet the ISTAART-AA MBI criteria or had an MBI-C score = 0. Detailed demographic data of all participants are listed in Table 1.

**TABLE 1:** Demographic data of the participants.

	**MBI group**	**Control group**	***P*-value**
Total number (*n*)	32	38	
Gender (man/female)	3/5	10/9	0.151^a^
Age (years)	67.31 ± 6.58	66.26 ± 7.34	0.795^b^
Education (years)	9.31 ± 1.54	11.94 ± 2.42	0.000^*^^b^
MMSE score	28.19 ± 1.20	28.78 ± 0.78	0.022^*^^b^
GDS score	10.19 ± 3.72	2.63 ± 1.44	0.000^*^^b^
SAS score	51.19 ± 4.80	28.26 ± 2.67	0.000^*^^b^
MBI-C total score	10.38 ± 1.45	0.00 ± 0.00	0.000^*^^b^
Decreased drive/motivation	3.56 ± 1.71	0.00 ± 0.00	–
Affective/emotional dysregulation	5.12 ± 1.54	0.00 ± 0.00	–
Impulse dyscontrol	1.81 ± 1.68	0.00 ± 0.00	–
Social inappropriateness	0.00 ± 0.00	0.00 ± 0.00	–
Abnormal thoughts/perception	0.00 ± 0.00	0.00 ± 0.00	–

*^a^Chi-square test.*

*^b^Non-parametric test (Mann–Whitney *U* test).*

*P* < 0.05 was considered significant.

*Represent significant difference.

*MMSE, Mini-Mental State Examination; GDS, Geriatric Depression Scale; SAS, Self-rating Anxiety Scale; MBI-C, Mild Behavioral Impairment checklist.*

### Magnetic Resonance Imaging Data Acquisition and Preprocessing

T1-weighted structural MRI scans were acquired with the MAGNETOM Skyra 3.0T at the Department of Radiology, Huadong Hospital, Shanghai (repetition time/echo time = 220/2.46 ms, slice thickness = 5 mm, spacing = 1.0 mm, FOV = 23.00 cm, total slices = 22). T2-weighted imaging, diffusion-weighted imaging, fluid-attenuated inversion recovery were also acquired. Previous studies reported that high white matter (WM) hyperintensities burden were associated with the increased risk of dementia and cognitive decline (Habes et al., 2016; Soldan et al., 2020). Thus we assessed WM hyperintensities according to the Fazekas scale (Fazekas et al., 1989) and participants who had a Fazekas scale score of ≥2 were excluded to remove the confounding effect of this factor on the brain network analysis. However, no participants were excluded. Image preprocessing was performed using CAT12^1^ based on SPM12^2^, which was described in detail in a previous study (Li et al., 2020). Briefly, all structural images were segmented into GM, WM, and cerebrospinal fluid images. High-dimensional DARTEL normalization was applied to normalize and modulate the GM images. The voxel size of the modulated GM images was 1.5 × 1.5 × 1.5 mm.

### Gray Matter Structural Covariance Network Construction

We used the Automated Anatomical Labeling template to parcellate the brain into 90 cortical and subcortical regions of interest (ROIs), as was done in previous studies (Yao et al., 2010). Regional GM volumes of each ROI were extracted and corrected for age, gender, education and mean overall GM volume by a linear regression analysis (He et al., 2007). Then, a 90 × 90 correlation matrix for each group was constructed by calculating the Pearson correlations between the corrected regional GM volumes. Adjacency correlation matrices were binarized and generated at a range of densities (0.05–0.5, with an interval of 0.01).

### Global Network Parameters

To characterize the topological organization of the structural covariance networks, widely used global network parameters were applied in this study, including the clustering coefficient, characteristic path length, small-world index, global efficiency, and local efficiency. Briefly, the clustering coefficient of a node is defined as the ratio of the number of existing connections between the direct neighbors of the node to the maximal possible number of connections between them. The clustering coefficient of a network is the average of the clustering coefficients across all nodes in the network, which can measure the network segregation of the brain. The characteristic path length of a network refers to the average shortest path lengths between all pairs of nodes in the network and is a measure of network integration. The normalized clustering coefficient (gamma) and normalized path length (lambda) are defined as the ratios of the clustering coefficient and the characteristic path length, respectively, of the brain network to those of 1,000 matched random networks (He et al., 2008). The small-world index (sigma) is computed as the ratio of gamma to lambda (Humphries and Gurney, 2008). A sigma value greater than 1 is a hallmark of a small-world network, reflecting an optimized balance between segregation and integration in the network. The global efficiency is calculated by taking the inverse of the harmonic mean of the shortest path lengths across pairs of nodes in a network, showing the global efficiency of the parallel information transfer in the network. The global efficiency of a subgraph composed of the nearest neighbors of a node is referred to as the local efficiency of that node. The local efficiency of a network is the average of the local efficiencies across all nodes.

### Regional Network Parameters

Nodal betweenness centrality is used to describe the regional characteristics of the structural covariance networks. It is given by the taking fraction of all the shortest paths passing through a node in the network, capturing the influence of a node on the information flow between other nodes in the network, and was normalized by the mean network betweenness centrality for group comparison.

### Network Hubs

Network hubs are essential for integrating diverse information sources and supporting efficient information communication. In the present study, a node whose nodal betweenness centrality was at least one standard deviation higher than the average network betweenness centrality was defined as a hub (Li et al., 2020).

### Statistical Analyses

Non-parametric permutation tests with 1,000 repetitions were performed to test intergroup differences in global and regional network parameters (He et al., 2008; Zhang et al., 2019). The corrected GM volumes of all participants were randomly reassigned into two new groups in each permutation. A corresponding binarized matrix in each randomized group was constructed using the same range of densities as in the real brain networks. The network parameters and the intergroup differences for each randomized group were calculated at each density to generate a permutation distribution of differences under the null hypothesis. For each network parameter, the actual difference between patients with MBI and the controls was placed in its corresponding permutation distribution to obtain the significance level. The significance level for group differences in the global and regional network parameters was set at *p* < 0.05 and after false discovery rate correction was carried out for multiple comparisons. The Brain Connectivity Toolbox was used to calculate the network parameters and detect the structural covariance network differences between groups (Rubinov and Sporns, 2010). BrainNet Viewer was employed for network visualization (Xia et al., 2013).

## Results

### Global Network Analysis

**FIGURE 1:**
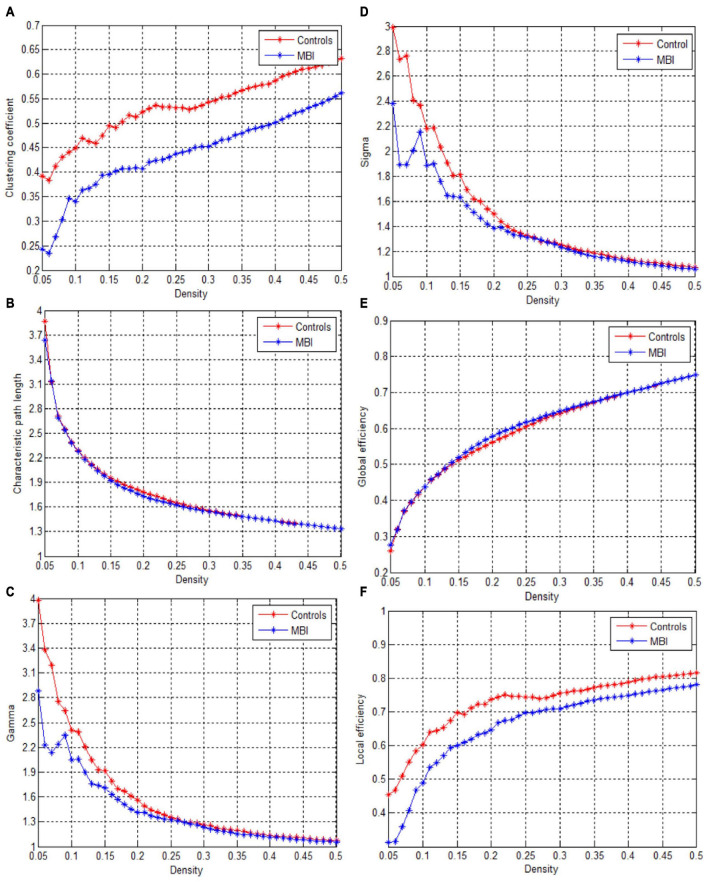
Changes in global network parameters as a function of network density. **(A)** Clustering coefficient, **(B)** Characteristic path length, **(C)** Gamma, **(D)** Sigma value, **(E)** Global efficiency, **(F)** Local efficiency in normal controls (controls) and MBI patients.

**FIGURE 2:**
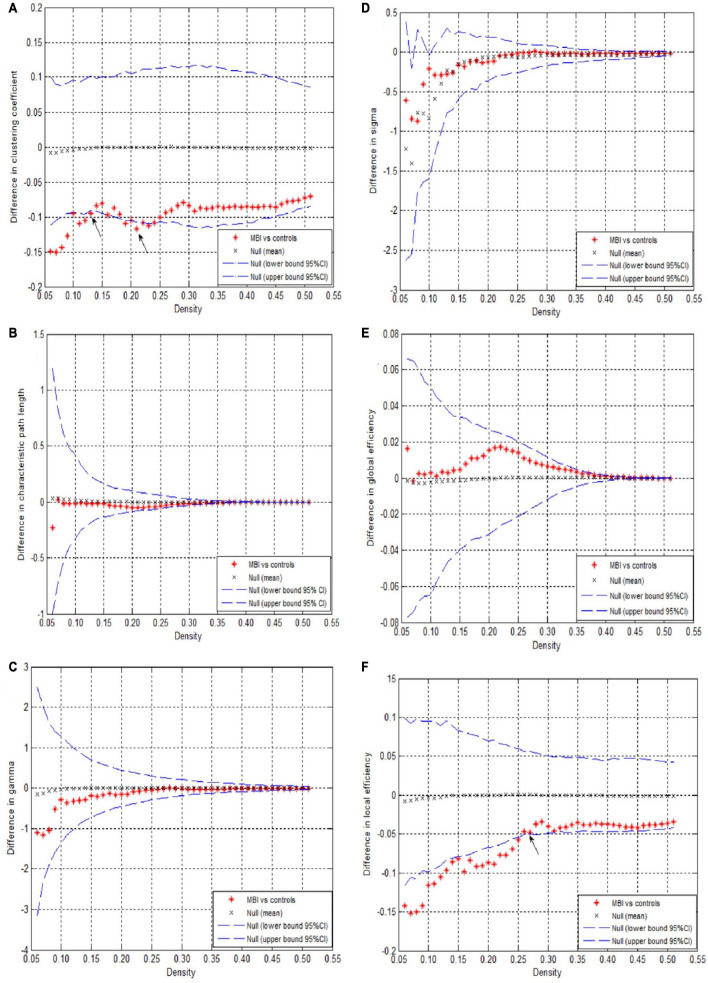
Differences between normal controls and MBI patients in global network parameters as a function of network density. The 95% confidence interval and group differences in **(A)** Clustering coefficient, **(B)** Characteristic path length, **(C)** Gamma, **(D)** Sigma value, **(E)** Global efficiency, **(F)** Local efficiency. The * red marker denotes the difference between normal controls and MBI patients; the * signs lying outside of the confidence intervals indicate the density where the difference is significant at *P* < 0.05. The positive values indicate MBI patients > controls and negative values indicate MBI patients < controls. The arrows represents the critical point where there is a significant difference between the two groups.

This study showed that the structural covariance networks of both groups followed a small-world topology across the range of densities, as evidenced by sigma values greater than one (Figure 1D). As shown in Figure 1, the clustering coefficient (Figure 1A), local efficiency (Figure 1F), gamma value (Figure 1C), and sigma value (Figure 1D) in the MBI group were lower than those in the control group across densities ranging from 0.05 to 0.5 (clustering coefficient and local efficiency) and from 0.05 to 0.26 (gamma and sigma values). The characteristic path length (Figure 1B) and global efficiency (Figure 1E) of both groups were similar. Significant differences (*p* < 0.05) in the clustering coefficient at several network densities (Figure 2A, arrows) and in the local efficiency values at 0.05 < density < 0.26 were detected between groups (Figure 2F, arrows). No significant intergroup differences were detected in the characteristic path length (Figure 2B), gamma value (Figure 2C), sigma value (Figure 2D), or global efficiency (Figure 2E).

### Regional Network Analysis

**FIGURE 3:**
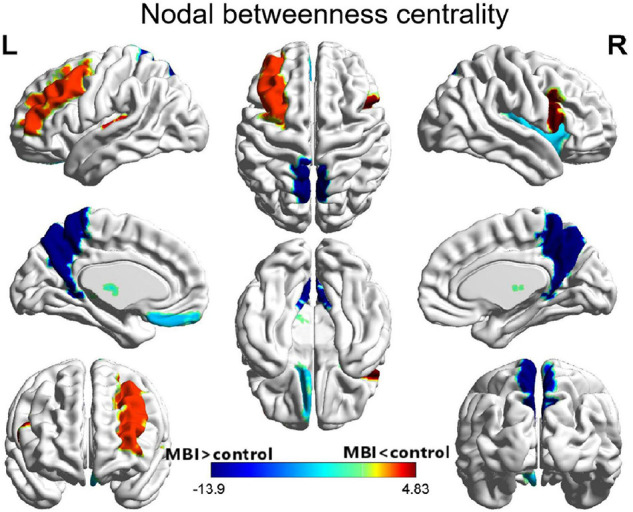
Differences between MBI group and normal control group in nodal betweenness centrality. Regions that showed significant differences between both groups in regional network topology were presented by the AUC of full connectivity mapped on ICBM152 surface template. The color bar represents log(1/*p*-value). The hot colors in the color bar represent regions that have significantly higher nodal betweenness centrality in the normal controls than in the MBI group, while cold color indicates regions with significantly higher nodal betweenness in the MBI group than in the normal controls. L, left; R, right.

Compared with the normal controls, patients with MBI demonstrated a significant decrease in nodal betweenness centrality in the left middle frontal gyrus, right inferior frontal gyrus (opercular part), and left Heschl gyrus. Conversely, some regions, including the left gyrus rectus, right insula, bilateral precuneus, and left thalamus, in the MBI group showed a significant increase in nodal betweenness centrality. However, of all these regions, only the bilateral precuneus survived after correcting for multiple comparisons (*p* < 0.05) (Figure 3 and Supplementary Table S1).

### Network Hub Analysis

**FIGURE 4:**
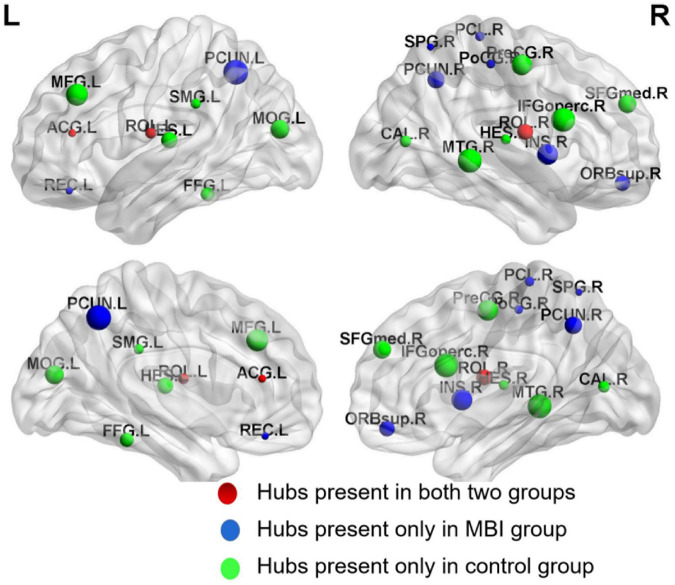
Network hubs in normal controls and MBI patients. Red color represents common network hubs in both groups, blue color represents hubs only in MBI group, and green color represents hubs only in control group.

A different number and distribution of network hubs were found between the MBI group and the control group. Specifically, 14 network hubs in the control group (Figure 4), i.e., the right precentral gyrus, left middle frontal gyrus, left inferior frontal gyrus (opercular part), bilateral Rolandic operculum, right medial superior frontal gyrus, left anterior cingulate and paracingulate gyri, right calcarine fissure and surrounding cortex, left middle occipital gyrus, left fusiform gyrus, left supramarginal gyrus, bilateral Heschl gyrus, and right middle temporal gyrus, and 11 network hubs in the MBI group, i.e., the right superior frontal gyrus (orbital part), left middle frontal gyrus, right Rolandic operculum, left gyrus rectus, right insula, left anterior cingulate and paracingulate gyri, right postcentral gyrus, right superior parietal gyrus, bilateral precuneus, and right paracentral lobule, were identified. Three network hubs were common to both groups: the bilateral Rolandic operculum and the left anterior cingulate and paracingulate gyri (Figure 4).

## Discussion

In this study, we investigated whether the topological organization of structural covariance networks was disrupted in patients with MBI compared with that in healthy controls. The patients with MBI had significantly decreased clustering coefficients at several network densities and local efficiency at densities ranging from 0.05 to 0.26, indicating decreased local segregation. No significant differences in the characteristic path length, gamma value, sigma value, or global efficiency were detected. In addition, both decreased and increased nodal betweenness centrality and a different number and distribution of network hubs were observed in patients with MBI compared with the normal controls. These findings provide novel insights into the altered topological organization in the structural covariance networks of patients with MBI.

Previous studies demonstrated that the brain networks of healthy individuals have an economical small-world topology (Liao et al., 2017). In line with these studies, we found that the GM structural covariance networks of both patients with MBI and controls showed a small-world topology, which provided further evidence supporting the notion that small-world topology is a fundamental organizational principle of structural brain networks.

The results of the global network analysis showed significantly lower clustering coefficients and local efficiency in patients with MBI compared with controls, indicating alterations in network topological properties in patients with MBI, consistent with previous studies (Yao et al., 2010; Menon, 2011; Zhao et al., 2012; Reijmer et al., 2013; Brier et al., 2014). The clustering coefficient measures the extent of local interconnectivity in a network, and local efficiency reflects the efficiency of the exchange of information between subgraphs. Lower clustering coefficient and local efficiency indicate decreased network segregation in patients with MBI, which is in accordance with previous findings that the local efficiency of the network was decreased in patients with AD and that the clustering coefficient was lower in cognitively normal participants who harbored the AD biomarker pathology (Zhao et al., 2012; Brier et al., 2014).

The present study showed significantly decreased nodal betweenness centrality in the left middle frontal gyrus, right inferior frontal gyrus (opercular part), and left Heschl gyrus in patients with MBI. These regions partly overlap the frontoparietal control network (FPCN), which encompasses regions across the frontal and parietal cortices. The FPCN plays a critical role in flexible information processing and goal-related behavior (Nico et al., 2007). Our results were in agreement with findings that altered FPCN connectivity was associated with affective symptoms in MCI (Munro et al., 2015; Joo et al., 2017). Previous studies have also reported NPS-related atrophy, reduced metabolism and perfusion, and WM abnormalities in the frontal and parietal cortices in MCI and AD patients (Ota et al., 2012; Trzepacz et al., 2013; Delrieu et al., 2015).

The present study also showed significantly increased nodal betweenness centrality in several brain regions in patients with MBI, including the left gyrus rectus, right insula, bilateral precuneus, and left thalamus. Moreover, the differences in the bilateral precuneus survived after correcting for multiple comparisons, which is consistent with previous studies showing increased nodal betweenness centrality of the precuneus in MCI and AD groups (Yao et al., 2010) and children with epilepsy (Li et al., 2020).

The precuneus, as a key region of the default mode network (DMN) (Utevsky et al., 2014), plays a crucial role in self-referential processing and episodic memory retrieval and emotion regulation (Fransson and Marrelec, 2008; van Buuren et al., 2010). The increased nodal betweenness centrality in the precuneus in patients with MBI implies the increased influence of the precuneus on information flow in the network. However, previous studies have shown amyloid deposition (Dickerson et al., 2009), reduced cerebral glucose metabolism (Tripathi et al., 2014) and decreased functional connectivity (Balthazar et al., 2014) in the precuneus in patients with AD. Drzezga et al. (2011) employed a multimodal imaging method in non-demented subjects and found that a higher amyloid burden correlated with lower whole-brain connectivity and metabolism, particularly in the posterior cingulate cortex/precuneus. Other studies using resting-state functional MRI (fMRI) demonstrated decreased resting-state activity and connectivity of the precuneus in patients with depression (Liu et al., 2017; Li et al., 2018; Dutta et al., 2019). A recent study found a significant correlation between higher rumination levels and decreased connectivity strength degree of the right precuneus in patients with major depressive disorder (Jacob et al., 2020). These results seem to contrast with our results, one possible explanation for which is that the increased nodal betweenness centrality in the precuneus might indicate hyperactivity of the precuneus, resulting in an impaired ability to synchronize with the rest of the network and leading to the apparent reduced connectivity. Since the present study was based on the structural covariance analysis of structural MR images, the functional connectivity of the precuneus could not be analyzed. In the future, resting-state fMRI analysis is needed to further confirm this hypothesis.

The hubs in both groups in the present study were predominantly located in regions of the association cortex, in accordance with previous studies (He et al., 2008; Drzezga et al., 2011; Bai et al., 2012) and evidencing the pivotal role of the association cortex in receiving convergent information in human cortical networks and combining this information to create emergent psychological properties (Goldman-Rakic, 1988). Three common hubs between the two groups were identified, including the left anterior cingulate cortex (ACC) and bilateral Rolandic operculum. The ACC is one of the crucial limbic structures in emotional self-control and behavioral and cognitive processing (Allman et al., 2001). Converging evidence has shown that decreased GM volume, perfusion or metabolism in the ACC were found in patients with AD exhibiting NPSs (Rosenberg et al., 2015). Another study found aberrant ACC connectivity during information processing in schizophrenia (White et al., 2010). The ACC was identified as one of the common hubs, indicating its pivotal role in the brain network.

The patients with MBI and normal controls also differed in the number and distribution of network hubs. More paralimbic cortices, including the right superior frontal gyrus (orbital part), left gyrus rectus, and right insula, were identified as hubs in the patients with MBI than in the normal controls. The insula, a core region of the salience network (SN) with extensive connections to many areas of the cortex and limbic system, receives and integrates various types of information, including motivational, emotional, and cognitive information, from cortical and subcortical regions and then relays this information to other regions to influence subsequent choice and action (Grupe and Nitschke, 2013). Dysfunction in the insula could lead to inappropriate behaviors. Previous studies have shown hyperactivation of the anterior and mid-insula across different anxiety disorders (Etkin and Wager, 2007; Qiao et al., 2017). Other studies using resting-state fMRI demonstrated increased insular functional connectivity associated with hyperactivity syndrome (i.e., agitation, irritability, aberrant motor behavior, euphoria, and disinhibition) (Balthazar et al., 2014) and decreased connectivity of the insula associated with apathy syndrome in patients with AD (Jones et al., 2019).

We also observed that the bilateral precuneus, which had a higher nodal centrality in patients with MBI, became a new hub region, which may reflect compensatory recruitment.

## Limitations

This study had some limitations that should be addressed. First, the sample size of our study was relatively small and thus may not reflect real cortical networks as accurately as possible. Second, this study built the structural covariance network at the group level, which is impossible to perform the correlation analysis between the network parameters and neuropsychological/behavioral scores. Third, in this study, GM structural covariance network was constructed based on clinical quality MRI, which may be not optimal for assessing GM structural covariance. However, Nowrangi et al. (2020) also utilized the clinical quality MRI to examine the interaction between structural brain volume measures and occurrence of neuropsychiatric symptoms (NPS) in outpatient memory clinic patients. Compared to other studies from research settings, We hope this study will serve as a validating first step in the eventual application of more sophisticated neuroimaging methods to study neural mechanisms underlying MBI. Future research from our group will implement inversion-prepared 3D T1-weighted sequences (e.g., MP-RAGE) to assess structural connectivity at single subject level and elucidate the relationship between network parameters and neuropsychological/behavioral scores in patients with MBI.

## Conclusion

The present study revealed the abnormal topology of the structural covariance networks in patients with MBI. Our findings offer new insights into the network dysfunctional mechanisms of MBI, and alterations in the topological patterns of structural covariance networks may serve as potential biomarkers for the early detection and diagnosis of patients with MBI at high risk of dementia. Future studies with data from additional imaging modalities, such as transcranial magnetic stimulation, DTI, and resting state-fMRI, are warranted to further explore this issue.

## Data Availability Statement

The raw data supporting the conclusions of this article will be made available by the authors, without undue reservation.

## Ethics Statement

This study was approved by the Ethics Committee of Huadong Hospital (2019K145), and informed consent was obtained from all participants. The patients/participants provided their written informed consent to participate in this study.

## Author Contributions

All authors listed have made a substantial, direct and intellectual contribution to the work, and approved it for publication.

## Conflict of Interest

The authors declare that the research was conducted in the absence of any commercial or financial relationships that could be construed as a potential conflict of interest.

## Publisher’s Note

All claims expressed in this article are solely those of the authors and do not necessarily represent those of their affiliated organizations, or those of the publisher, the editors and the reviewers. Any product that may be evaluated in this article, or claim that may be made by its manufacturer, is not guaranteed or endorsed by the publisher.

## Footnotes

1
http://dbm.neuro.uni-jena.de/cat/

2
http://www.fil.ion.ucl.ac.uk/spm
