# Supplementary material for: Aberrant Topological Patterns of Structural Covariance Networks in Cognitively Normal Elderly Adults With Mild Behavioral Impairment
Source: Front Neuroanat. 2021 Sep 29;15:738100. doi: 10.3389/fnana.2021.738100 (PMC8511486; doi:10.3389/fnana.2021.738100)
Supplement: Supplementary file 1 [file Table_1.DOCX]

Table S1 Regions showing abnormal nodal betweenness centrality or node degree in MBI patients as compared with normal controls

|  | Brain regions | P value,  uncorrected | Brain regions | P vale,  uncorrected |
| --- | --- | --- | --- | --- |
|  | MBI group > normal control group |  | MBI group < normal control group |  |
| **Nodal betweenness centrality** |  |  |  |  |
|  | Gyrus rectus-Left | 0.006 | Middle frontal gyrus- Left | 0.044 |
|  | Thalamus-Left | 0.017 | Inferior frontal gyrus, opercular part-Right | 0.008 |
|  | Precuneus-Left | 0 | Heschl gyrus- Left | 0.015 |
|  | Precuneus-Right | 0 |  |  |
|  | Insula-Right | 0.009 |  |  |
| **Nodal degree** |  |  |  |  |
|  | Gyrus rectus-Left | 0.001 | Inferior frontal gyrus, opercular part-Right | 0.021 |
|  | Gyrus rectus- Right | 0.025 | Supramarginal gyrus -Left | 0.029 |
|  | Precuneus- Left | 0.001 | Heschl gyrus- Left | 0.002 |
|  | Precuneus - Right | 0.027 |  |  |
|  | Thalamus-Left | 0.015 |  |  |
